# Unravelling the skin of the nurse shark: A morphological description of the placoid scales of *Ginglymostoma cirratum*

**DOI:** 10.1111/jfb.70345

**Published:** 2026-02-26

**Authors:** Danilo P. Lima, Aline. N. Poscai, Laura F. Mianutti, Nathalie. M. R. de Abreu, Jade Medeiros, Marcus V. G. Araújo, Frederico. B. de Sousa, João Paulo C. B. da Silva

**Affiliations:** ^1^ Departamento de Sistemática e Ecologia, Centro de Ciências Exatas e da Natureza Universidade Federal da Paraíba João Pessoa Brazil; ^2^ Centro Nacional de Pesquisa e Conservação de Mamíferos Carnívoros (CENAP) Instituto Chico Mendes de Conservação da Biodiversidade (ICMBio) Atibaia Brazil; ^3^ Programa de Pós‐Graduação em Odontologia, Centro de Ciências da Saúde Universidade Federal da Paraíba João Pessoa Brazil; ^4^ Departamento de Zoologia, Instituto de Biociências Universidade de São Paulo, Cidade Universitária São Paulo Brazil; ^5^ Departamento de Morfologia, Centro de Ciências da Saúde Universidade Federal da Paraíba João Pessoa Brazil

**Keywords:** Chondrichthyes, dermal denticles, Elasmobranchii, mineralization, Orectolobiformes

## Abstract

*Ginglymostoma cirratum*, commonly known as the nurse shark, is a nocturnally active benthic shark, often found in western and eastern Atlantic waters. Although this shark has been well explored in many biological aspects, few studies have thoroughly examined the morphology of its dermal denticles (or placoid scales). The dermal denticles are teeth‐like structures present in the skin of sharks and play an important role in the interaction of these fishes with their environment. In the present study, we describe the morphological variation of dermal denticles throughout the body of the nurse shark and discuss the putative functional roles of the morphologies uncovered. Twenty‐four skin samples were extracted from different regions of the body and were analysed using a microtomography and a stereoscope. The terminology was standardized based on previous morphological descriptions of denticles. A total of 10 variations were identified, ranging from denticles with few or no ridges with a lozenge‐shaped crown to denticles with multiple ridges bearing diamond‐ or leaf‐shaped crowns. The morphology of the scales supports a primary function associated with abrasion strength, as previously suggested by other authors. Also, the location and distribution of ridged denticles may indicate a secondary function associated with hydrodynamics. The diagnosis of a second species within the genus, *Ginglymostoma unami*, based on differences in dermal denticle morphology was also discussed. The Unami nurse shark was described as having denticles with a distinct morphology and a greater number of ridges than in *G. cirratum*. However, we suggest that *G. cirratum* and *G. unami* have denticles with a similar morphology and ridge counts.

## 1 INTRODUCTION

The nurse shark is one of the only two recognized species within the genus *Ginglymostoma* and is included in the family Ginglymostomatidae (Orectolobiformes). *Ginglymostoma cirratum* (Bonnaterre 1788) is a nocturnally active shark, often found in rocky and coral reef complexes at depths of up to 130 m in shallow tropical waters off the western and eastern Atlantic (Ebert et al., 2021; Weigmann, 2016). There is an extensive debate regarding the size of maturity and maximum size for *G. cirratum*. Current estimates indicate that the size at birth may be around 270 mm total length (TL), reaching 2300 mm TL at maturity and a maximum size of nearly 3000 mm TL (Castro, 2000, 2011; Ebert et al., 2021; Weigmann, 2016). Additionally, till recently, the genus was considered monotypic. Nevertheless, a new species, the Unami nurse shark *Ginglymostoma unami* Del Moral‐Flores et al., 2015 has been recently described.

The nurse shark is a relatively well‐studied species, having been the subject of research involving its general biology, functional morphology, genetics and population genetics (e.g., Castro, 2000; Garla et al., 2017; Karl et al., 2012; Luer et al., 1990; Motta et al., 2008; Robinson & Motta, 2006; Silva & Carvalho, 2015; Silva & Vaz, 2023). However, few studies have investigated the variation in placoid scales along the body of the nurse shark (e.g., Dillon et al., 2017; Ferrón & Botella, 2017; Raschi & Tabit, 1992).

The placoid scales, or dermal denticles, are teeth‐like structures that cover the body of elasmobranchs. They are primarily composed of dentine and are externally covered by enameloid, having a mineralized base that serves as a site of attachment to the skin of the fish (Atkinson & Collin, 2012; Paig‐Tran et al., 2022). Dermal denticles are normally present around the entire body of sharks, but their distribution may vary across elasmobranch taxa, being scarce in most orders of rays and completely absent in electric rays (Torpediniformes) (Cappetta, 2012). Additionally, they can vary in shape, size and number along the body of the same individual, being associated with different functional roles (Atkinson & Collin, 2012; Dillon et al., 2017; Paig‐Tran et al., 2022; Raschi & Tabit, 1992).

Due to the variable morphology of denticles, some authors have described different morphotypes based on their functionality. Currently, five general functions are recognized: generalized functions, drag reduction, defence, abrasion strength and luminescence (Reif, 1985). This variation is directly associated with the behaviour and activity pattern of species. Active pelagic sharks exhibit predominantly hydrodynamic drag‐reduction denticles, whereas demersal sharks display predominantly denticles adapted to abrasion strength (Dillon et al., 2017; Raschi & Tabit, 1992; Reif, 1985).

The denticles of the nurse shark were briefly described in Raschi and Musick (1984), Reif (1985) and Raschi and Tabit (1992). However, their primary goal was to comparatively describe the morphology of the denticles across various shark taxa, discussing their implications for hydrodynamics, protection, bioluminescence and other functional roles. Ferrón and Botella (2017) proposed a generalized denticle morphology for *G. cirratum* and other species of sharks and compared them to the odontodes found in Thelodonts.

The most complete morphological variation in placoid scales of the nurse shark was reported by Dillon et al. (2017), who analysed 14 different skin samples. However, similar to previous studies, their research did not focus solely on the nurse shark, but included other shark taxa, aiming to correlate dermal denticle morphology with its ecological roles to test whether dermal denticles can serve as reliable tools for accessing extinct and extant populations. Nevertheless, the specific morphological variation in placoid scales of *G. cirratum* was not completely addressed and explored.

In this context, this study aims to describe the morphological variation in placoid scales along the body of *G. cirratum*, using images generated by micro‐CT scan, stereo microscopy and scanning electron microscopy (SEM). We also discuss the putative functional implications of placoid scales' relation to their variable morphology.

## 2 MATERIALS AND METHODS

### 2.1 Specimens

**FIGURE 1:**
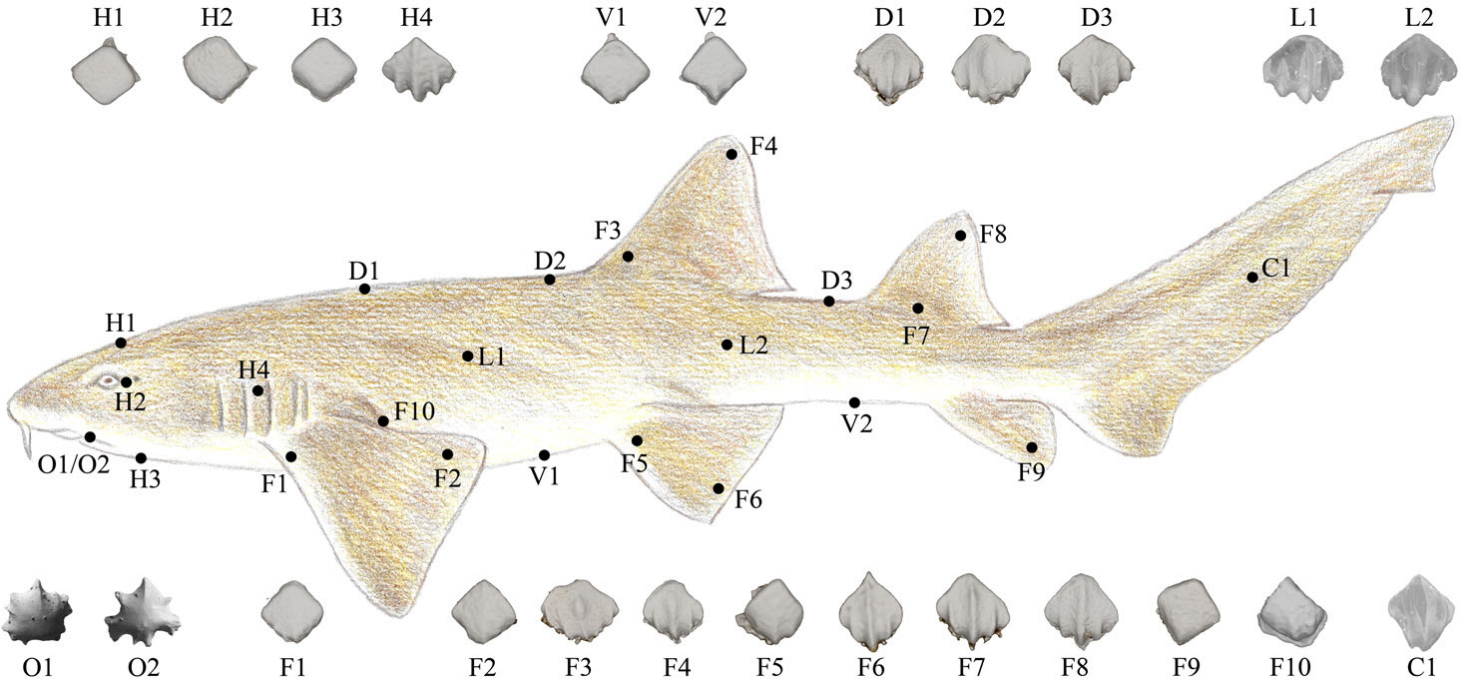
Distribution of denticles sampled from *Ginglymostoma cirratum*. H1, H2, H3 and H4 = head type denticles; V1 and V2 = ventral type denticles; D1, D2 and D3 = dorsal type denticles; L1 and L2 = lateral type denticles; F1, F2, F3, F4, F5, F6, F7, F8, F9 and F10 = fin‐type denticles; C1 = caudal‐type denticles; O1 and O2 = oropharyngeal‐type denticles. Images from micro CT‐scan: H1–H4, V1–V2, D1–D3 and F1–F10. Images from light microscopy: L1–L2 and C1. Images from SEM: O1 and O2.

The specimens of *G. cirratum* examined in this study are housed in the following institutions: Universidade Federal da Paraíba (UFPB) (UFPB 14343, subadult female, 1068 mm precaudal length [PRC]; UFPB 1972, juvenile female, 468 mm PRC, 669 mm TL) and the Elasmobranch Research Laboratory at Universidade Estadual Paulista (UNESP) (IB/CLP UNESP uncat., female embryo, 265 mm TL). Measurements followed Compagno (1984), and all specimens were preserved in 70% ethanol. Twenty‐four skin samples were taken from different regions of the body of each specimen for analysis (Figure 1), and all samples were preserved in microtubes with 70% ethanol. An average of 15 denticles per sample was analysed.

### 2.2 Micro CT‐scan

Nineteen samples (H1, H2, H3, H4, D1, D2, D3, V1, V2, F1, F2, F3, F4, F5, F6, F7, F8, F9 and F10) were scanned using a Skyscanner 1172 microtomograph (Bruker) at the Laboratório de Microscopia e Imagem Biológica (LAMIB/UFPB). The scanning parameters were as follows: rotation step 0.60°, frame 4, random movement 10, medium resolution (2000 × 1332 pixels), no filter, 60kv–167uA. The subsequent reconstructions were performed with the following settings: smoothing 4; rings 3; beam‐hardening 15. Output: 0.075174–0.258727. The resulting images were reconstructed using Amira (version 5.3.3).

### 2.3 Light and SEM

Samples from the oropharyngeal cavity (O1 and O2) were dehydrated in increasing series of ethanol (70%–100%), then dried on a critical point device (Balzers CPD 030) and metalized with a gold layer with a metalizer device (EMITECH K550). Samples were later photographed using a scanning electron microscope (LEO 440 Zeiss) at the Museu de Zoologia da Universidade de São Paulo (MZUSP). The resulting images were edited using PhotoScape X software (version 4.1.1.). The remaining images (L1, L2 and C1) were obtained through photographs taken using a Leica M205A microscope from the Paulo Young Laboratory of Marine Invertebrates (LIPY/ UFPB).

### 2.4 Dermal denticle terminology

A standardized terminology was proposed for the morphological descriptions of denticles based on Reif (1985), Poscai et al. (2021, 2022) and Vaz et al. (2023). Samples were separated in different body regions, including the head, dorsal, ventral, lateral trunk portions and fins (paired and unpaired). Posteriorly they were classified in different types based on their morphological similarities. The terminology used for the denticles was standardized to start the morphological descriptions from their dorsal portion (crown) towards their ventral portion (basal root). The terms star, lozenge, diamond and leaf shape were employed to describe the shape of the crown of denticles. Star shape refers to the unique nearly star‐shaped crown from the dorsal view of the oropharyngeal cavity. The lozenge term refers to a more uniform shape, where the four sides of the structure are nearly or similar in extension. Diamond and leaf shapes refer to sides that are asymmetrical and bear different extensions. Diamond shape refers to asymmetrical shapes with straightened posterior margins, and leaf refers to curved posterior margins.

### 2.5 Morphological descriptions of denticles

The morphological descriptions of dermal denticles were organized primarily by their location in the body of the nurse shark. Consequently, all denticle types presented in results reflect this form of organization. This organization was chosen considering that denticles of different portions of the body may be similar in morphology (e.g., samples C1 and F4). Additionally, our objective was to describe denticles along the body and not to group them based on the morphology presented. Furthermore, all morphological variations which are organized based on the morphological similarities and differences in denticles are presented in Table 1.

**TABLE 1:** Measurements of the crown samples.

Denticle sample	CL (mm)	CW (mm)
O1	0.18	0.20
O2	0.22	0.24
H1	0.80	0.78
H2	0.78	0.82
H3	0.57	0.72
H4	0.51	0.57
D1	1.13	1.16
D2	1.24	1.33
D3	0.82	0.88
V1	0.60	0.66
V2	0.87	0.85
L1	0.86	0.98
L2	0.92	1.11
F1	0.64	0.67
F2	0.56	0.55
F3	1.23	1.52
F4	0.65	0.58
F5	0.42	0.49
F6	0.60	0.51
F7	0.73	0.73
F8	0.65	0.66
F9	0.59	0.57
F10	0.48	0.50
C1	0.71	0.64

*Note*: All of the measures are referent to the denticles presented in the corresponding figures.

Abbreviations: Cl, crown length; Cw, crown width.

### 2.6 Ethics statement

All specimens examined in the present study were previously housed in ichthyological collections. No specimens were harmed or collected by any of the authors during the course of this research.

## 3 RESULTS

### 3.1 Terminology

**FIGURE 2:**
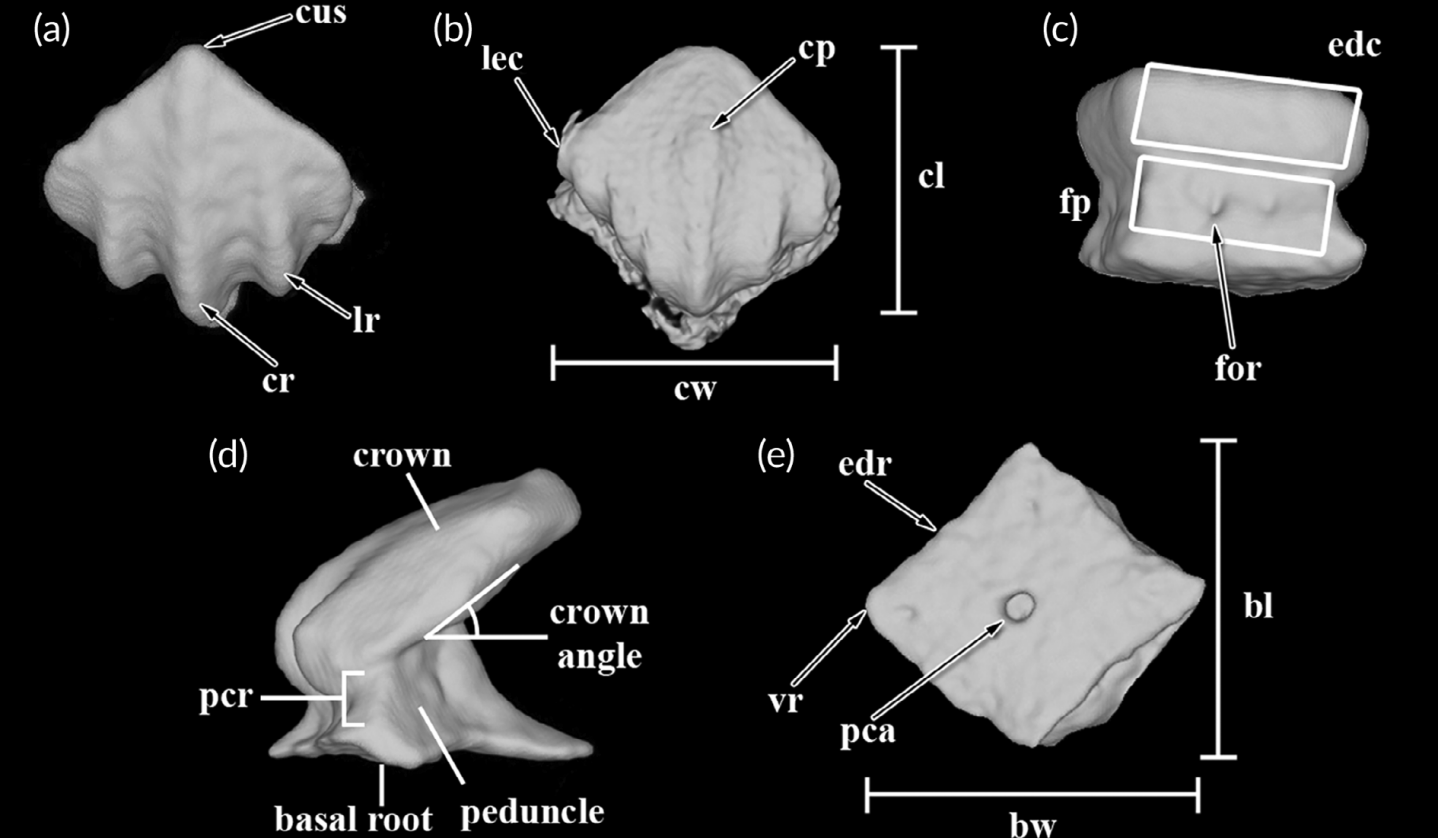
Terminology (see Terminology section in Results). (a, b) Dorsal view of denticles. (c) Frontal view of denticle. (d) Lateral view of denticle. (e) Ventral view of denticles. Bl, base length; bw, base width; cl, crown length; cw, crown width; cp, central peak; cr, central ridge; cus, cusp; edc, edge of the crown; edr, edge of the basal root; for, foramen; fp, face of the peduncle; lr, lateral ridge; pca, pulp cavity; pce, peduncle crest; vc, vertex of the crown; vr, vertex of the peduncle. In (a, b, e, f) anterior to bottom; In (c) anterior to front; In (d) anterior to left (micro CT‐scan).

- Crown (Poscai et al., 2021, 2022; Reif, 1985; Vaz et al., 2023): Visible portion of the denticle (Figure 2);
- Ridges (Poscai et al., 2021, 2022; Reif, 1985; Vaz et al., 2023): Protuberances along the crown surface, normally oriented in the anterior–posterior axis. They can vary in shape, size and extension;
- Central ridge – cr (Vaz et al., 2023): The centremost ridge of the crown is typically prominent. In the present study, some denticles are observed to have two central ridges;
- Lateral ridges – lr (Vaz et al., 2023): Ridges positioned laterally to the central ridge;
- Central peak – cp (present paper): Correspond to the most elevated portion of the central ridge;
- Lateral extensions of the crown – lec (present paper): Most lateral extensions of the crown;
- Cusps – cus (Poscai et al., 2021, 2022; Reif, 1985; Vaz et al., 2023): Acute tip portion of the crown. Vaz et al. (2023) define a cusp as an extremely prominent and visibly pointed structure. In contrast, Poscai et al. (2021, 2022) classify less pointed peaks as cusps in cases where Vaz et al. (2023) do not. Here we consider any peaked portion that achieves an open angle of 90° or less from the dorsal view of the denticle as a cusp;
- Edge of the crown – edc (present paper): Region comprising the edge portions of the crown;
- Crown angle (Vaz et al., 2023): Angle formed between the anteroposterior portion of the crown and the anteroposterior portion of the peduncle.
- Crown length – cl (Vaz et al., 2023): Anteroposterior extension of the crown;
- Crown width – cw (Vaz et al., 2023): Extension across the most lateral portions of the crown;
- Peduncle (Poscai et al., 2021, 2022; Reif, 1985; Vaz et al., 2023): The portion of the denticle that connects the crown with the basal root. The peduncle is somewhat lozenge shaped and usually bears four surfaces (face of the peduncle) that have small foramina;
- Face of the peduncle – fp (present paper): Each of the four edges immediately under the crown that extends in between each ridge of the peduncle;
- Peduncle ridge – pcr (present paper): Ridges that originate in the anterior portion of a vertex and that extend across the peduncle reaching the lower portion of the crown. They are typically four in number, intercalating between the faces of the peduncle. The anterior ridge of the peduncle may also merge with the central cusp;
- Foramen – for (Vaz et al., 2023): Openings found occasionally on the face of the peduncle. It's synonymous to Base Neck‐Canal in Reif (1985);
- Basal root (Poscai et al., 2021, 2022; Reif, 1985; Vaz et al., 2023): Portion of the denticle attached to the skin of the animal. It normally has four angular edges;
- Base length – bl (Vaz et al., 2023): Antero‐posterior extension of the base of the root;
- Base width – bw (Vaz et al., 2023): Lateral extension of the base of the root;
- Edge of the basal root‐edr (adapted from Vaz et al., 2023): Margin of the basal root that interconnects the vertices. All denticles in this study are present in sets of four;
- Vertex of the root – vr (present paper): The most extended portions of the root. There are four in all samples (two lateral, one anterior and one posterior);
- Pulp cavity – pca (Poscai et al., 2021, 2022; Reif, 1985; Vaz et al., 2023): A cavity present in all denticles that opens into the ventral portion of the basal root.

### 3.2 Descriptions

Crown length (CL) and crown width (CW) of the following denticle descriptions are presented in Table 1.

**FIGURE 3:**
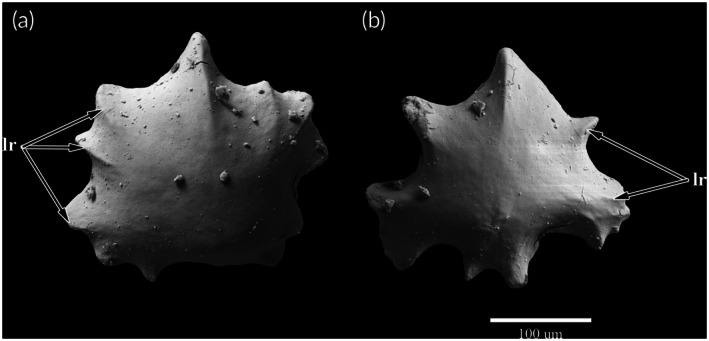
Oropharyngeal type cavity (IB/CLP UNESP uncat. female, 265 mm total length [TL]). (a) O1 upper view from ventral oral denticle. (b) O2 upper view from dorsal oral denticle. In (a, b) anterior to bottom (scanning electron microscopy).

- 1. Oropharyngeal cavity type: (Figure 3, samples O1, ventral; O2, dorsal)

The denticles of the oropharyngeal cavity (O1‐O2) have a nearly star‐shaped crown with four to eight ridges surrounding it. The most posteriormost ridge is more developed in relation to the lateral ones, with nearly two times their size. The crown width (**cw**) is greater than the crown length (**cl**). A central peak (**cp**) occurs in some oral denticles at the centre of the crown. Additionally, the ridges are oriented towards the oesophagus. Peduncle and root morphology were not analysed.

- 2. Head type 1: (Figure 4.1, samples H1, dorsal; H2, spiracular)

**FIGURE 4:**
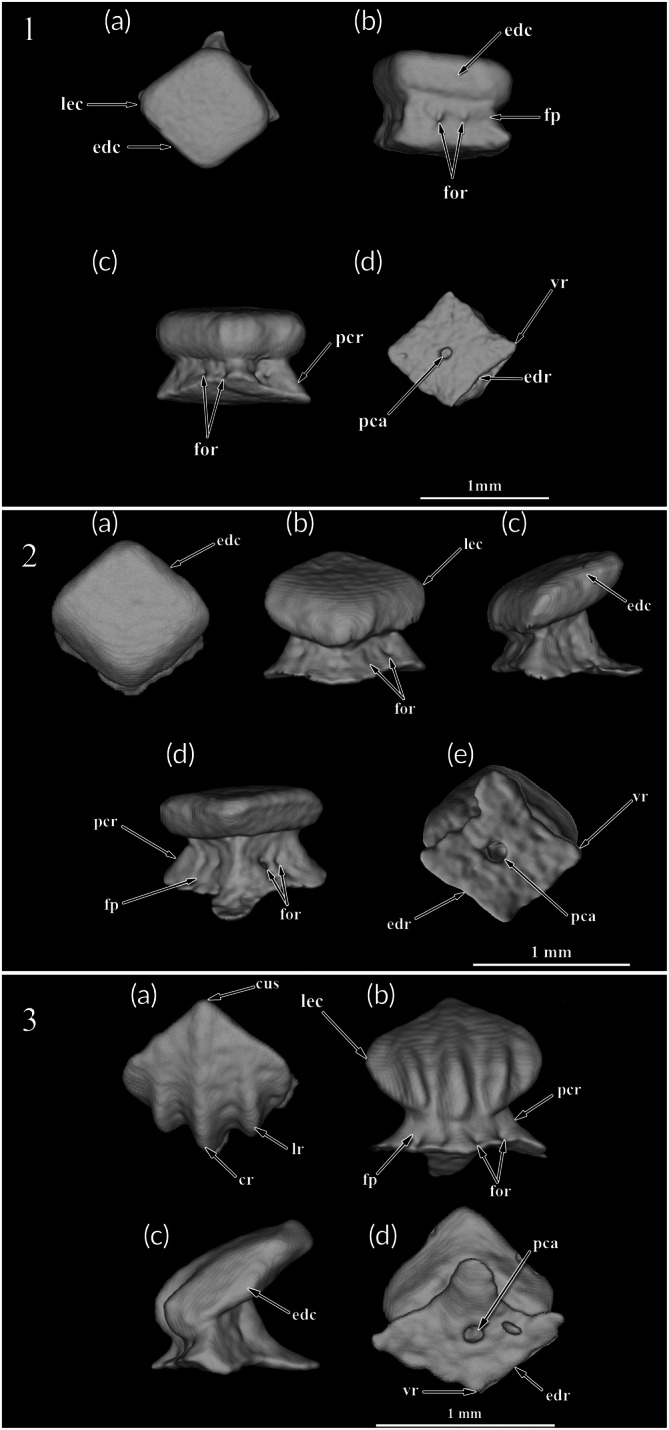
1 = Head type 1 (UFPB 14343 female, 1068 mm PCR). (1a) H1 dorsal view. (1b) H1 frontal view. (1c) H1 posterior view. (1d) H2 ventral view. In (1a, 1d) anterior to bottom; In (1b) anterior to front; In (1c) anterior to back. 2 = head type 2 denticles (UFPB 14343 female, 1068 mm PCR). (2a) H3 dorsal view. (2b) H3 frontal view. (2c) H3 lateral view. (2d) H3 posterior view. (2e) H3 ventral view. In (2a, 2e) anterior to bottom; In (2b) anterior to front; In (2c) anterior to left; In (2d) anterior to back. 3 = Head type 3 (UFPB 14343 female, 1068 mm PCR). (3a) H4 dorsal view. (3b) H4 frontal view. (3c) H4 lateral view. (3d) Ventral view. In (3a, 3d) anterior to bottom; In (3b) anterior to front; In (3c) anterior to left (micro CT‐scan).

The crown is predominantly lozenge‐shaped. **cw** and **cl** vary little among denticles. The crown has a smooth surface and does not bear cusps or ridges. Some denticles may have an oval‐shaped crown. In the dorsal and ventral regions of the head, the crown surface and the peduncle have the same width, with no crown angle to the peduncle. All edges of the crown (**edc**) are thick and convex.

The peduncle of the head type 1, along with all dorsal (D1, D2 and D3), ventral (V1 and V2) and fin‐type 5 (F3), is the most robust, having almost the same width as the crown and basal root. It is lozenge‐shaped, following the same morphology of the crown. It has four surfaces (**fp**) and four ridges (**pcr**) in between them (one anterior, one posterior and two lateral). All peduncle ridges originate at the vertices and extend dorsally to the ventral portion of the crown. Each surface of the peduncle (**fp**) bears two small foramina (**for**).

The basal root is also predominantly lozenge‐shaped and has a pulp cavity (**pca**) that pierces its central portion. Each margin is nearly straight and devoid of curvature. The base length (**bl**) is slightly greater than the base width (**bw**), a condition that is more evident in H2 denticles. Both anterior edges of the basal root (**edr**) are straight, whereas the posterior ones are slightly concave. The anterior and the lateral vertices of the root (**vr**) form an angle of nearly 90°, whereas the posterior vertex is more acute in H1. In H2 denticles, the anterior and lateral vertices have a smaller angle.

- 3. Head type 2: (Figure 4.2, sample H3, ventral)

The crown has a smooth surface that lacks cusps or ridges, with an overall lozenge shape, a condition also observed in the dorsal trunk and spiracular regions. The anterior edges of the crown (**edc**) form a slightly convex angle, whereas the posterior ones are more convex. The crown width (**cw**) is slightly greater than the crown length (**cl**), and the lateral extensions of the crown (**lec**) are anterior in relation to its midportion, giving a more elongated aspect to its posterior portion. Except for a small posterior part of the crown, most of its area is attached to the peduncle. There is a crown angle, when compared to type 1 head denticles, with its posterior portion slightly ascending from the peduncle (H1 and H2).

The peduncle is less robust than that of type 1 head denticles, but also lozenge‐shaped. Additionally, the anterior surfaces of the peduncle are slightly shorter than the posterior ones, which are also slightly concave.

- 4. Head type 3: (Figure 4.3, sample H4, interbrachial)

The crown is leaf‐shaped and has no great distinction between crown length (**cl**) and crown width (**cw**). The crown is leaf‐shaped and has no great distinction between crown length (**cl**) and crown width (**cw**). The lateral extensions of the crown (**lec**) have the same position as type 2 head denticles (H3). The crown may have one to three ridges, one central (**cr**) and two lateral (**lr**), and a single subtle cusp (**cus**). Additionally, the anterior edges of the crown (**edc**) are continuous with the peduncle. The central ridge originates on the anterior peduncle ridge (**pcr**) and extends posteriorly over the crown, reaching the tip of the cusp. Lateral ridges are restricted to the crown and originate on its anterior edges, extending posteriorly to the midportion of the crown surface. The cusp forms an angle that is slightly less than 90°. Only the anterior half of the crown is attached to the peduncle, whereas the posterior half remains free. Additionally, the crown is oblique in relation to the peduncle.

The peduncle is narrower when compared to type 1 head denticles (H1 and H2). Each anterior surface of the peduncle is pierced by two foramina (**for**), whereas each posterior surface is pierced by a single foramen. The peduncle is also narrower in comparison to the crown and the root.

The basal root is star‐shaped and has a proportion similar to type 2 head denticles. The anterior and lateral angular edges are smaller than the posterior ones. The base length (**bl**) is shorter than base width (**bw**). The posterior margins of the basal root (**edr**) are concave, more evidently than other head denticles. The anterior and lateral vertices of the root (**vr**) are acute, and the posterior rounded. The aperture of the pulp cavity (**pca**) may be slightly larger than other head denticles.

- 5. Dorsal type 1: (Figure 5.1, samples D1, dorsal; D2, dorsal trunk)

**FIGURE 5:**
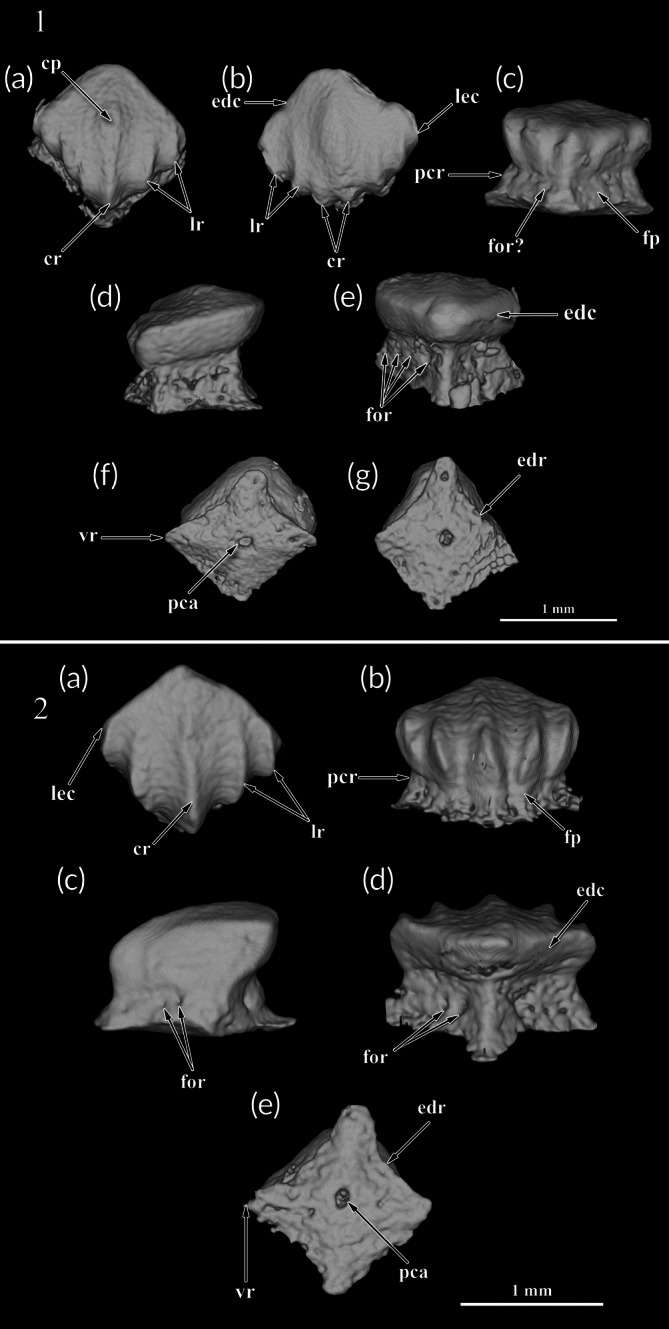
1 = Dorsal type 1 denticles (UFPB 14343 female, 1068 mm PCR). (1a) D1 dorsal view. (1b) D2 dorsal view. (1c) D1 frontal view. (1d) D1 lateral view. (1e) D2 posterior view. (1f) D1 ventral view. (1g) D2 ventral view. In (1a, 1b, 1f, 1g) anterior to bottom; In (1c) anterior to front; In (1d) anterior to left; In (1e) anterior to back. 2 = Dorsal type 2 denticles (UFPB 14343 female, 1068 mm PCR). (2a) D3 dorsal view. (2b) D3 frontal view. (2c) D3 lateral view. (2d) D3 posterior view. (2e) D3 ventral view. In (2a, 2e) anterior to bottom; In (2b) anterior to front; In (2c) anterior to left; In (2d) anterior to back (micro CT‐scan).

The crown is diamond‐shaped and has five to nine ridges on its anterior portion. There is no great distinction between crown length (**cl**) and crown width (**cw**). All ridges originate on the anterior edge of the crown (**edc**). Some denticles may bear two central ridges (**cr**), that extend posteriorly over almost the entire area of the crown surface, ending in a central peak (**cp**) that is located centrally, and clearly discernible in dorsal view. Lateral ridges (**lr**) also extend posteriorly but do not reach the midportion of the crown surface, remaining mostly on its anterior half. All denticles are devoid of cups. Most of the crown area is connected to the peduncle, forming a slight crown angle. Denticles in D1 have only a small posterior portion that is free from the peduncle.

The peduncle resembles the overall description of type 1 head denticles (H1 and H2). Some denticles have four foramina (**for**) on each face of the peduncle. The basal root is predominantly star‐shaped with a smooth surface. Both anterior edges of the basal root (**edr**) are straight, whereas both posterior edges are concave, similar to previous samples. The posterior edges of D1 denticles of the basal root are more concave when compared to those of D2. The relationship between base length (**bl**) and base width (**bw**) is irregular, with base length exceeding base width in some denticles but not in others. The anterior half of the basal root has a vertex (**vr**) with an angle of approximately 90°, which is larger when compared to the posterior one. The aperture of the pulp cavity (**pca**) is small, occupying a small portion of the total area of the root, a condition similar to type 1 head denticles.

- 6. Dorsal type 2: (Figure 5.2, sample D3, dorsal between first and second dorsal fins)

The crown has an overall diamond shape. The lateral extensionsof the crown (**lec**) are located posterior to the midline. There is no great distinction between crown length (**cl**) and crown width (**cw**). The crown surface has five ridges: a central one (**cr**) and four lateral (**lr**). All ridges originate on the anterior edge of the crown (**edc**) and extend posteriorly over nearly all of the crown surface. Denticles are devoid of cusps. Most of the crown is connected to the peduncle, with a small posterior portion being free, similar to what is observed in D1 denticles.

The peduncle resembles the general morphology of type 1 head denticles (H1 and H2), but bears two foramina (**for**) on each face. The basal root is star‐shaped, similar to what was described for D1 denticles.

**FIGURE 6:**
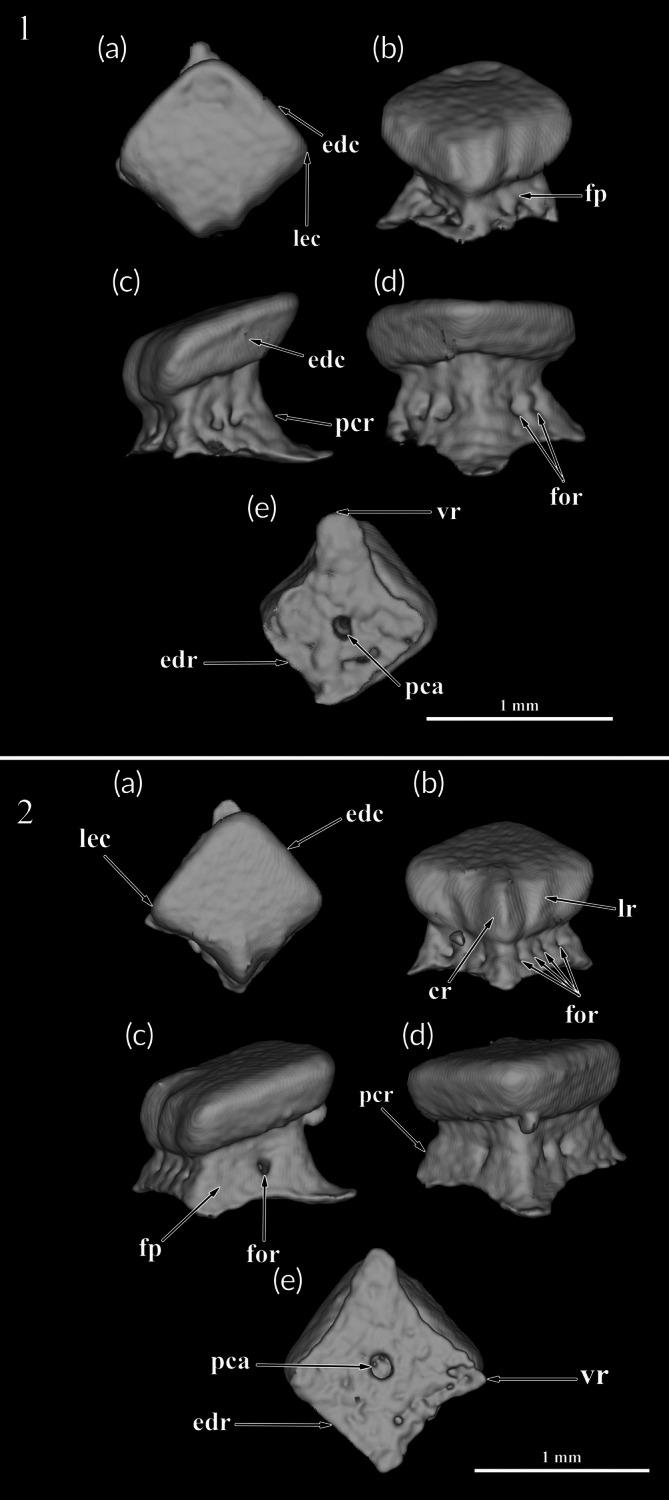
1 = Ventral type (V1 denticles; UFPB 14343 female, 1068 mm PCR). (1a) V1 dorsal view. (1b) V1 frontal view. (1c) V1 lateral view. (1d) V1 posterior view. (1e) V1 ventral view. In (1a, 1e) anterior to bottom; In (1b) anterior to front; In (1c) anterior to left; In (1d) anterior to back. 2 = Ventral type denticles (V2 denticles; UFPB 14343 female, 1068 mm PCR). (2a) V2 dorsal view. (2b) V2 frontal view. (2c) V2 lateral view. (2d) V2 posterior view. (2e) V2 ventral view. In (2a, 2e) anterior to bottom; In (2b) anterior to front; In (2c) anterior to left; In (2d) anterior to back (micro CT‐Scan).

- 7. Ventral type: (Figure 6, samples V1, abdomen; V2, inferior between pelvic and anal fins)

The crown is lozenge‐shaped. Both lateral extensions (**lec**) are located on the midportion of the crown. The crown surface is smooth and lacks ridges. Part of the denticles in V2 have small ridges restricted to the anterior edge of the crown (**edc**), which may vary from three to five, whereas V1 denticles have indiscernible ridges. There are no major differences between the crown length (**cl**) and crown width (**cw**). All denticles are devoid of cusps. Most of the crown area is connected to the peduncle with only a small posterior portion being free, similar to D1 type 1 dorsal denticles. There is a crown angle similar to type 2 head denticles (H3).

The peduncle is robust and lozenge‐shaped, following the general morphology of type 1 head denticles (H1 and H2). In V1, all faces of the peduncle (**fp**) have two foramina (**for**). In V2, each anterior face bears four foramina, whereas each posterior face bears a single one.

The basal root is star‐shaped in V1 and predominantly lozenge‐shaped in V2. The anterior and posterior portions are slightly different, similar to dorsal type 1 (D1 and D2) and dorsal type 2 (D3) samples. Both anterior edges of the basal root (**edr**) are straight, whereas the posterior edges are concave. The posterior edges of the basal root in V1 denticles are evidently more concave than in V2. Some denticles in V1 also have a posterior vertex of the root (**vr**) more distant from the aperture of the pulp cavity (**pca**) than the anterior vertex. The aperture of the pulp cavity is small, similar to type 1 head denticles (H1 and H2).

**FIGURE 7:**
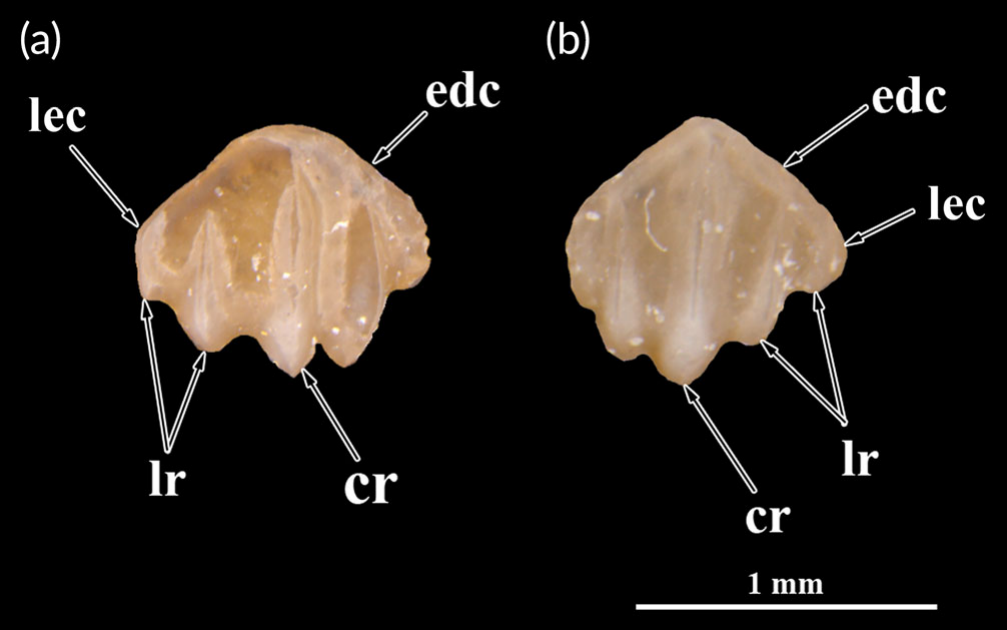
Lateral type denticles (UFPB 14343 female, 1068 mm PCR). (a) L1 dorsal view. (b) L2 dorsal view. In (a, b) anterior to bottom (light microscopy).

- 8. Lateral type: (Figure 7, samples L1, lateral trunk between insert of pectoral and pelvic fins; L2, lateral trunk between posterior base of first dorsal and pelvic fins)

The crown is diamond‐shaped. Refer to type 2 dorsal denticles (D3) for descriptions of the crown, peduncle and root.

- 9. Fin type 1: (Figure 8.1, sample F1, anterior base of the pectoral fin)

**FIGURE 8:**
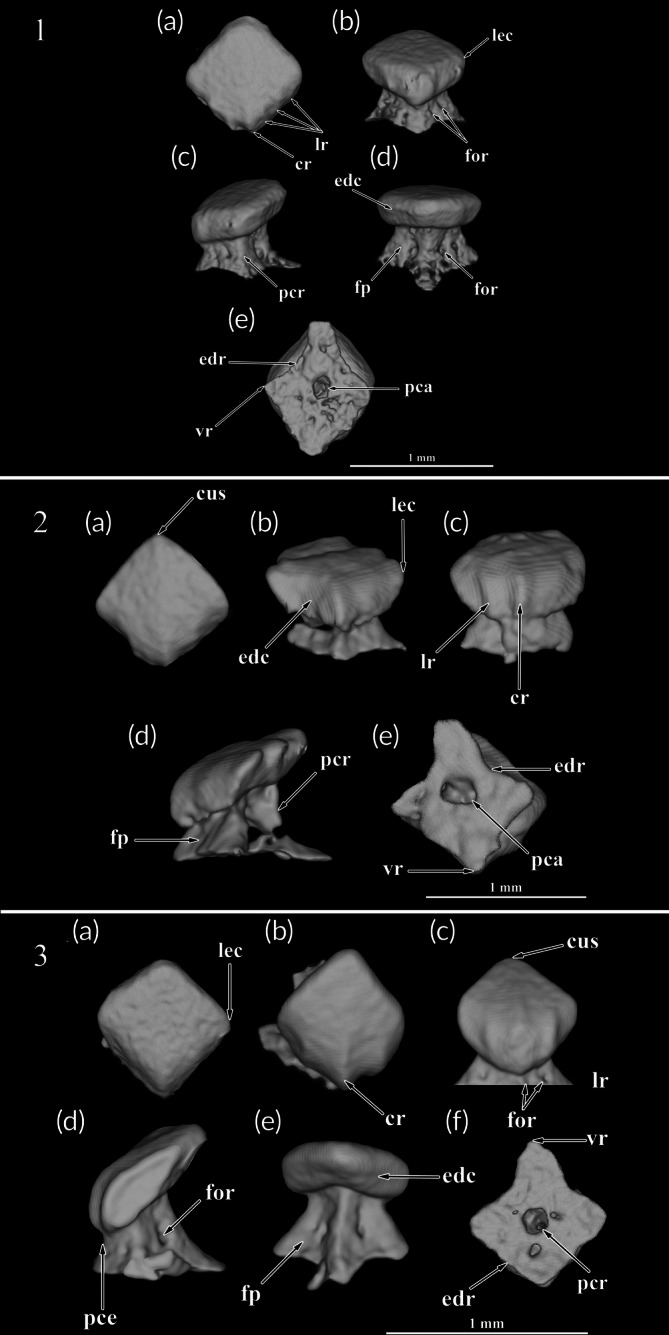
1 = Fin type 1 denticles (UFPB 14343 female, 1068 mm PCR). (1a) F1 dorsal view. (1b) F1 frontal view. (1c) F1 lateral view. (1d) F1 posterior view. (1e) F1 ventral view. In (1a, 1e) anterior to bottom; In (1b) anterior to front; In (1c) anterior to left; In (1d) anterior to back. 2 = Fin type 2 denticles (UFPB 14343 female, 1068 mm PCR). (2a) F2 dorsal view. (2b) F2 frontal view. (2c) F2 frontal view. (2d) F2 lateral view. (2e) F2 ventral view. In (2a, 2e) anterior to bottom; In (2b, 2c) anterior to front; In (2d) anterior to left. 3 = Fin type 3 denticles (UFPB 14343 female, 1068 mm PCR). (3a) F9 dorsal view. (3b) F5 dorsal view. (3c) F5 frontal view. (3d) F5 lateral view. (3e) F5 posterior view. (3f) F9 ventral view. In (3a, 3b, 3f) anterior to bottom; In (3c) anterior to front; In (3d) anterior to left; In (3e) anterior to back (micro CT‐scan).

The shape of the crown varies between a lozenge and a diamond shape throughout the sample. Distinctions between crown length (**cl**) and crown width (**cw**) are asymmetric. Some denticle crowns are wider than long, whereas others express similar proportions. The denticles on this region are devoid of cusps. The crown is nearly smooth, with few denticles bearing small incipient ridges restricted to the anterior edge of the crown (**edc**). The number of ridges ranges from three to seven, with only a central ridge (**cr**). Denticles may have ridges restricted to the anterior edge of the crown, and the central ridge eventually extends posteriorly over almost all of the crown surface. Most of the crown is attached to the peduncle, whereas a small posterior portion, comprising the cusp, remains free, similar to type 2 head denticles (H3). The crown angle is similar to type 2 head denticles (H3).

The peduncle is lozenge‐shaped, resembling the general morphology of type 1 head denticles (H1 and H2). It has two foramina (**for**) in each of its anterior faces (**fp**) and one in each posterior face. The basal root is star‐shaped, similar to what was observed in type 1 dorsal denticles (D1). The base length (**bl**) and base width (**bw**) are similar. There may be slight differences in the diameter of the aperture of the pulp cavity (**pca**) among denticles.

- 10. Fin type 2: (Figure 8.2, sample F2, posterior inner margin of the pectoral fin)

The crown is diamond‐shaped. Distinctions between crown length (**cl**) and crown width (**cw**) are asymmetric following the same description in F1. Most of the lateral extensions (**lec**) across denticles are located on the anterior half of the crown. Denticles have a single subtle cusp (**cus**). The crown is smooth, bearing incipient ridges that are restricted to the anterior edge of the crown. Ridges can vary between three and five. Only one central ridge (**cr**) is present. Most of the crown area is connected to the peduncle, whereas only a small posterior portion, the cusp area, is free. The free area of the crown is longer than in F1. The crown is also obliquely oriented in relation to the peduncle, similar to the condition observed in head type 2 denticles (H2).

All faces of the peduncle (**fp**) are more concave than previous samples, specially the posterior ones. The ridges of the peduncle (**pcr**) are also more prominent. Each anterior face of the peduncle has two foramina (**for**). The basal root is star‐shaped with the same overall description for type 1 dorsal denticles (D1). There are not great differences in proportion between base length (**bl**) and base width (**bw**). The posterior edges of the basal root (**edr**) are evidently concave. The aperture of the pulp cavity (**pca**) is the largest compared to all previous samples.

- 11. Fin type 3: (Figure 8.3, samples F5, anterior base of the pelvic fin; F9, anal fin)

There is no great distinction between crown length (**cl**) and crown width (**cw**). The lateral extensions (**lec**) are located more anteriorly on the crown, similar to the condition described for type 3 samples of the head (H4). The denticles bear small ridges that vary from three to five, with one central (**cr**), and a discreet single cusp (**cus**). The ridges originate on the anterior edge of the crown (**edc**) and extend posteriorly almost reaching the midportion of the crown in F9 and reaching it in F5. Only the anterior portion of the crown is connected to the peduncle, whereas the posterior portion is not. The crown angle is similar to the condition observed in type 2 head denticles (H3).

The morphology of the peduncle is similar to type 2 fin denticles (F2). The anterior faces of the peduncle have two foramina (**for**) each, whereas the posterior faces of the peduncle (**fp**) have only one. The basal root is star‐shaped with the same characteristics of type 2 fin denticles.

- 12. Fin type 4: (Figure 9.1, sample F6, posterior outer margin of the pelvic fin)

**FIGURE 9:**
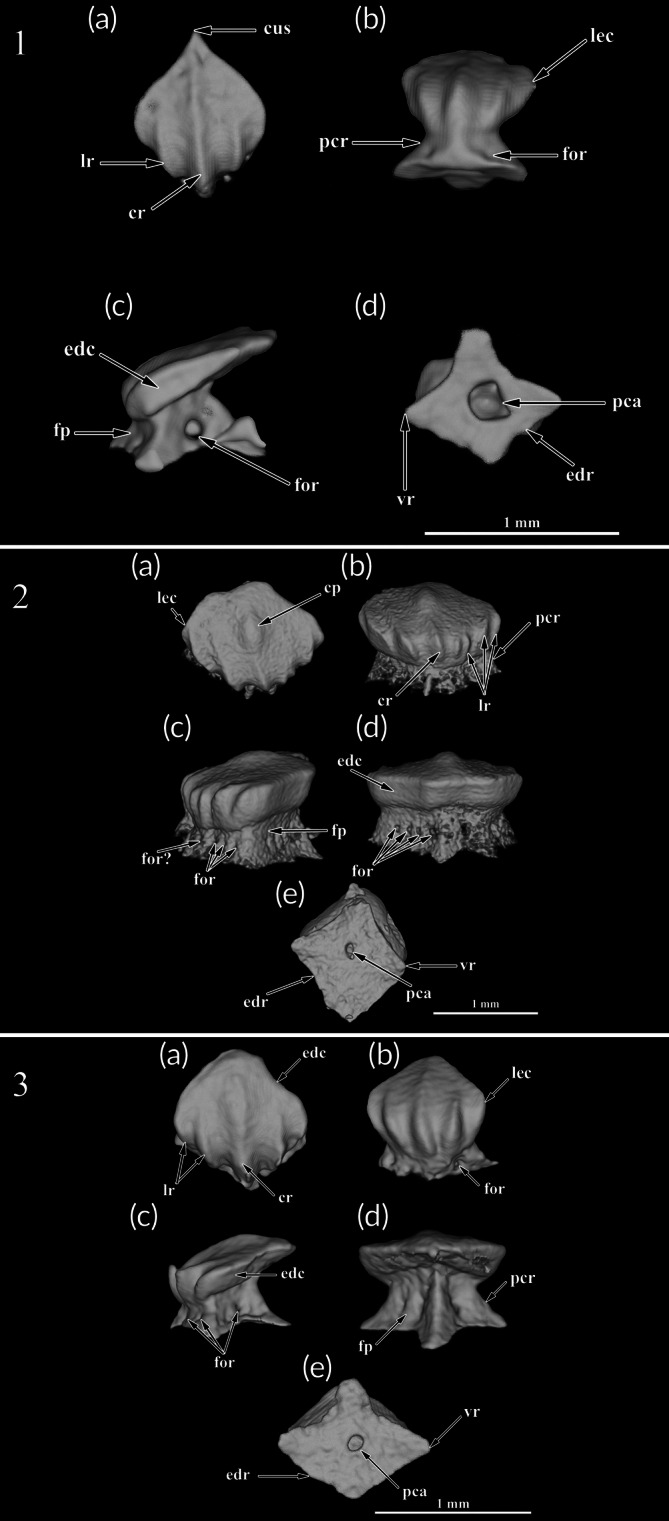
1 = Fin type 4 denticles (UFPB 14343 female, 1068 mm PCR). (1a) F6 dorsal view. (1b) F6 frontal view. (1c) F6 lateral view. (1d) F6 ventral view. In (1a, 1d) anterior to bottom; In (1b) anterior to front; In (1c) anterior to left. 2 = Fin type 5 denticles (UFPB 14343 female, 1068 mm PCR). (2a) F3 dorsal view. (2b) F3 frontal view. (2c) F3 lateral view. (2d) Posterior view. (2E) F3 ventral view. In (2a, 2e) anterior to bottom; In (2b) anterior to front; In (2c) anterior to left; In (2d) anterior to back. 3 = Fin type 6 denticles (UFPB 14343 female, 1068 mm PCR). (3a) F4 dorsal view. (3b) F7 frontal view. (3c) F4 lateral view. (3d) F8 posterior view. (3e) F7 ventral view. In (3a, 3e) anterior to bottom; In (3b) anterior to front; In (3c) anterior to left; In (3d) anterior to back (micro CT‐scan).

The crown is leaf‐shaped. The crown length (**cl**) is greater than crown width (**cw**) and both lateral extensions (**lec**) are located more anteriorly on the crown, similar to the condition described in type 3 denticles of the head (H3). The denticles have one central ridge (**cr**) and two lateral (**lr**). All ridges originate on the anterior edge of the crown (**edc**). Lateral ridges extend posteriorly slightly surpassing the midportion of the crown, whereas the central ridge reaches the cusp. All denticles have a single prominent cusp (**cus**). Only the anterior portion of the crown is connected to the peduncle, whereas the posterior portion is not, resembling the condition in type 3 fin denticles (F5 and F9). The crown angle is similar to the type 2 head denticles (H3).

The morphology of the peduncle is similar to type 2 fin denticles (F2). The anterior and posterior faces of the peduncle (**fp**) have one foramen (**for**) each. The basal root is star‐shaped with the same morphology of type 2 fin denticles. The base width (**bw**) is slightly greater than base length (**bl**). The diameter of the pulp cavity (**pca**) is relatively the largest found in all samples, occupying most part of the root area.

- 13. Fin type 5: (Figure 9.2, sample F3, anterior base of the first dorsal fin)

The crown is diamond‐shaped. The crown, peduncle and basal root have the same morphology of type 1 dorsal denticles (D2). The differences are in the number of ridges, which varies from seven to nine, and all faces of the peduncle (**fp**) have four foramina (**for**) each.

- 14. Fin type 6: (Figure 9.3, samples F4, distal posterior margin of the first dorsal fin; F7, mid base of the second dorsal fin; F8, distal posterior margin of the second dorsal fin)

The crown is leaf‐shaped. The crown length (**cl**) is slightly bigger than the crown width (**cw**), and both lateral extensions of the crown (**lec**) are positioned more anteriorly in relation to the mid‐line of the crown, similar to the condition described in type 3 head denticles (H4). The denticles have five ridges and no cusps. The central (**cr**) and lateral ridges (**lr**) originate on the anterior edge of the crown (**edc**) and extend posteriorly over all of the crown surface.

The peduncle has a morphology similar to type 2 fin denticles (F2). Each anterior face of the peduncle (**fp**) has two foramina (**for**), whereas each posterior face of the peduncle has one foramen. The basal root is star‐shaped and has the same characteristics of type 2 fin denticles (F2).

- 15. Fin type 7: (Figure 10.1, sample F10, pectoral axil)

**FIGURE 10:**
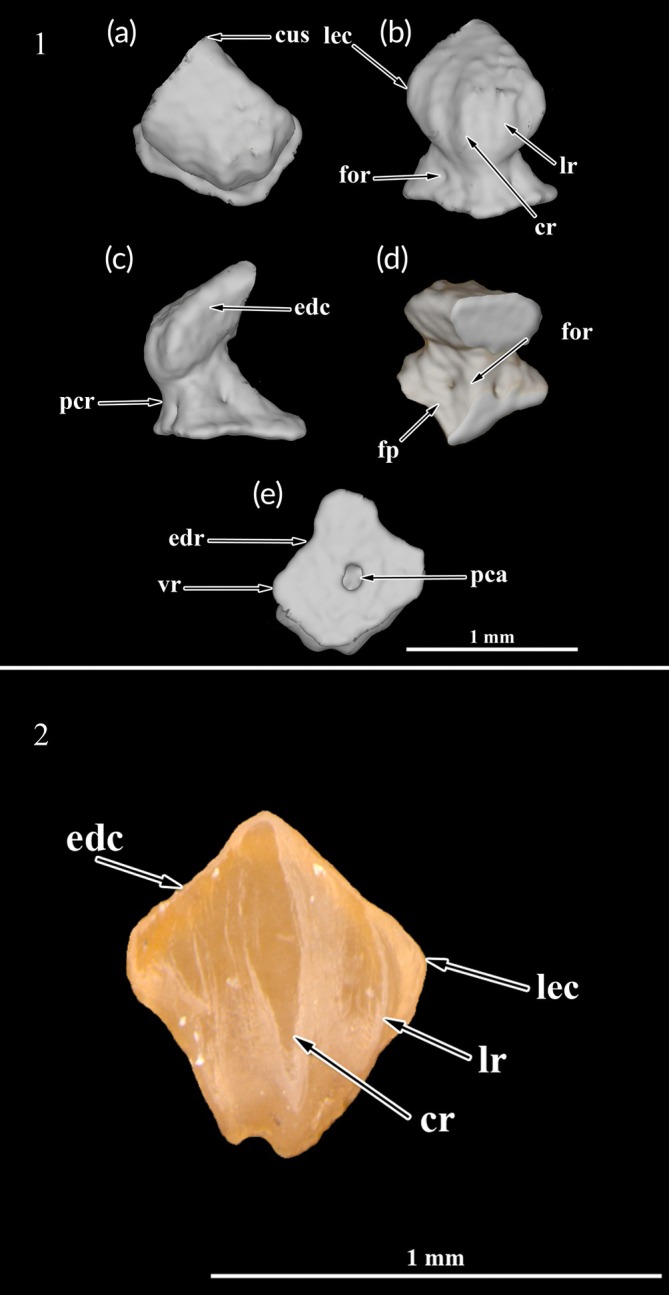
1 = Fin type 7 denticles (UFPB 14343 female, 1068 mm PCR). (1a) F10 dorsal view. (1b) F10 frontal view. (1c) F10 lateral view. (1d) F10 posterior view. (1e) F10 ventral view. In (1a, 1e) anterior to bottom; In (1b) anterior to front; In (1c) anterior to left; In (1d) anterior to back (micro CT‐Scan). 2 = Caudal‐type denticles (UFPB 1972 female, 669 mm TL). 2‐C1 dorsal view. In 2 anterior to bottom (light microscopy).

The crown is leaf‐shaped with both lateral extensions (**lec**) located more anteriorly on the crown. The posterior portion of the crown ends in a prominent single cusp (**cus**). There is no great distinction between crown length (**cl**) and crown width (**cw**). The crown has one central (**cr**) and two lateral (**lr**) ridges that originate on the anterior edge of the crown (**edc**) and extend posteriorly, reaching the midportion of the crown surface. Most of the anterior portion of the crown is connected to the peduncle, whereas its posterior‐half is not. The crown is not perpendicular to the peduncle, bearing a crown angle greater than type 3 head denticles (H4).

The peduncle follows the same morphology of type 2 fin denticles (F2), with one foramen (**for**) on each face of the peduncle (**fp**). The basal root has an irregular star shape. All vertices (**vr**) have a greater angle compared to the denticles of all other regions. Both anterior edges of the basal root (**edr**) are nearly straight, whereas the posterior ones are concave. The aperture of the pulp cavity (**pca**) has a diameter similar to type 2 fin denticles.

- 16. Caudal type: (Figure 10.2, sample C1, mid‐lateral region of the upper caudal lobe.)

The crown is diamond‐shaped. Descriptions for the crown, peduncle and root are similar to type 2 dorsal denticles (D3).

## 4 DISCUSSION

### 4.1 Morphological variability in dermal denticles

Dermal denticles play an important role in the interaction of sharks and their environment. Despite its variations, denticles may have similar morphologies even among taxa that are not closely related but, which may have similar behaviours (e.g., *Isurus oxyrinchus* Rafinesque), 1810 and *Sphyrna zygaena* (Linnaeus, 1758) (Reif, 1985). This indicates that denticle morphology is directly associated with shark behaviour or ecological guild (Dillon et al., 2017). Based on this premise, all the currently known morphotypes are classified based on their associated functionality for the body of shark across known taxa.

Of all five major functional groups described, the most frequently discussed is hydrodynamics (Raschi & Tabit, 1992). The denticles classified in hydrodynamics are normally the ones bearing ridges over the crown, and found in fast‐swimming sharks. It is commonly theorized that the presence of ridges would help in drag reduction by restricting turbulent flow of the boundary layer between the water and the skin of the animal (Dean & Bhushan, 2010). Abrasion strength denticles, on the contrary, are denticles commonly associated with benthic animals and responsible for protection against harsh surfaces, normally characterized by having a larger smooth crown area with few (or lacking) ridges (Reif, 1985). Dillon et al. (2017) subdivided this classification into two categories: abrasion strength and ridged abrasion strength.

One type of denticle in *G. cirratum* was evaluated by Reif (1978, 1985), and was described as having a thick scale crown with three parallel ridges and a strongly blunt rounded cusp. This type of denticle was collected from the ventral region between the first and the second dorsal fin (location B4 in Reif, 1985), and matches the same location and morphology of the denticles analysed in the present study for the sample D3. Despite the presence of ridges, denticle morphology was associated only with the abrasion strength functionality with no hydrodynamic contributions for this shark. Raschi and Musick (1986) and Raschi and Tabit (1992) evaluated denticles with a similar morphology, but instead they did not discard drag reduction contributions considering that models at the time were primarily based on maximum speed reached among species of sharks. The most comprehensive evaluation on denticle morphology of *G. cirratum*, conducted by Dillon et al. (2017), extracted 14 samples from this species. Five morphotypes were identified, and all subdivided between abrasion strength, ridged abrasion strength and generalized functions. Morphotype classifications followed Reif (1985) but lacked possible secondary functionalities and the functional differences between abrasion strength and ridged abrasion strength for denticles.

In the present work all five different morphological variations in denticles described in Dillon et al. (2017) in addition to the variation described in the previous morphological works of Reif (1978, 1985), Raschi and Musick (1986) and Raschi and Tabit (1992) were found (H1, H2, H3, H4, D3, V1, L1, L2, F2, F4, F5, F7, F8, F9 and C1). Additionally, five new morphologies for the denticles were described (O1, O2, F1, D1, D2, F3, F6, V2 and F10) with a total of 10 reported (see Table 2). The variation observed indicates evident morphological differences and the presence or absence of ridges.

**TABLE 2:** Morphological variation for dermal denticles in *Ginglymostoma cirratum* compiled from the literature combined with the ones found in the present work.

Morphological variation	Samples	Reported manuscripts	Figures
Variation 1	H1; H2; H3	Dillon et al., 2017	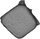
Variation 2	V1; V2	Dillon et al., 2017	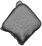
Variation 3	F2	Dillon et al., 2017	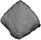
Variation 4	H4; L1; L2; Cl; D3; F4; F7; F8	Reif (1978, 1985), Raschi and Musick (1986); Raschi and Tabit (1992); Dillon et al., 2017	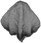
Variation 5	F5; F9	Dillon et al., 2017	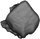
Variation 6	O1; O2	New	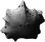
Variation 7	D1; D2; F3	New	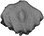
Variation 8	F1	New	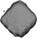
Variation 9	F6	New	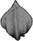
Variation 10	F10	New	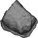

Most denticles are robust. Denticle crowns have edges (**edc**) reaching half of the vertical area of the peduncle, with the peduncle and root area nearly equal in size to the area of the crown. Also, crowns are not as obliquely oriented in relation to the peduncle, a condition also observed in not closely‐related sharks (e.g., *Carcharodon carcharias* (Linnaeus, 1758), *Mustelus canis* (Mitchill, 1815)) (Ankhelyi et al., 2018; Fath et al., 2024; Sayama et al., 2024). All head type denticles (Figure 4), with exception to head type 3 (Figure 4.3), have a lozenge‐shaped crown with no ridges and a robust peduncle that has more than three quarters of the total area of the crown. The ventral type and fin types 1, 2 and 3 denticles (Figures 6 and 8) are similar in morphology and bear a diamond‐shaped crown with incipient ridges that are either restricted to the anterior edge of the crown (edc) or extend all over the crown surface. Dorsal types 1 and 2, (Figure 5), lateral type (Figure 7) and caudal types (Figure 10.2) bear diamond‐shaped denticles with ridges extending almost or entirely over the crown surface. Furthermore, dorsal type 1 denticles (Figure 5.1) are the most robust found in this study and are the only samples along with fin type 5 denticles (Figure 9.2) that bear a central peak (cp). The denticles of fin types 4 and 7 (Figures 9.1 and 10.1) and of the head type 3 (Figure 4.3) have ridged leaf‐shaped crowns, with greater crown angles, and peduncles bearing two quarters of the total area of the crown. Denticles of the fin types 4 and 7 (pelvic and pectoral axil, respectively) are one of the most distinctive in crown morphology, with leaf‐shaped crowns (Figures 9.1 and 10.1). The most unusual morphology found for the denticles in this study was on the oropharyngeal type (Figure 3). Contrary to all other ridged denticles, they have an irregular star‐shaped crown with no distinct separation between its edges and with lateral ridges surrounding the crown.

The robust pattern of denticles described in this study was also reported for other closely related sharks, like *Nebrius ferrugineus* (Lesson 1831) in Reif (1985), with few denticles being morphologically similar to what is reported herein (type 2 fin denticles, Figure 8.2), although being from a different location (interbrachial tegument, sample H4 in Reif, 1985). Regarding the distribution of unridged and subtly ridged denticles (H1, H2, head type 1; H3, head type 2; V1, V2, ventral type; F1, fin type 1; F5, F9, fin type 3), a similar pattern is surprisingly reported for other unrelated taxa like *I. oxyrinchus*, *M. canis* and *C. carcharias* (Ankhelyi et al., 2018; Fath et al., 2024; Motta et al., 2012; Sayama et al., 2024), despite the different overall morphology of the denticle. In all three taxa, few denticles without ridges were found in the snout, whereas most anterior pectoral fin denticles had subtle ridges. However, an important difference is also present. All ventral denticles in other shark taxa are usually extremely ridged in contrast with the unridged denticles found in the ventral region of the *G. cirratum*.

### 4.2 Putative functionalities of dermal denticles

The robust pattern of the crown, peduncle and root described for most denticles found in this study indicates that the main function of placoid scales of *G. cirratum* may be to protect against abrasion. This is consistent with previous studies (Dillon et al., 2017; Ferrón & Botella, 2017; Raschi & Tabit, 1992; Reif, 1985) and also with the biology of the species. Considering that the nurse shark is normally associated with benthic rocky and coral reef complexes (Ebert et al., 2021), abrasion denticles are crucial for their protection against harsh surfaces. This is also in accordance with the results found by Schuitema et al. (2024), which shows that the nurse shark also has a thicker tegument that may also confer abrasion protection.

Despite the association of most denticles with abrasion strength, a great variation in crown morphology (lozenge, diamond and leaf‐shaped), together with the presence or absence of ridges, could also indicate a secondary functionality linked with hydrodynamics for part of the scales. As previously mentioned, most previous works disregard the contribution for hydrodynamics for two reasons: the benthic habit of the species, which is consistent with slow speed rates compared to other pelagic sharks, and the previous functional models for denticles applied by Bechert et al. (1985) and Reif (1985). Reif and collaborators developed a comparative model among different denticles of sharks in which ridge spacing confronted with the speed that the animal reaches determines if the denticle really has a role in drag reduction or not. In this context, only fast pelagic sharks like *I. oxyrinchus* would have denticles that could actually aid in drag reduction. Ridged denticles of sharks that reach less maximum swimming speed on the other hand would not impact in drag reduction, therefore not serving for this purpose.

Even though Reif's model is robust, all assumptions were made based on tests of models of denticles disregarding the free space that exists between the posterior portion of the crown and the posterior vertex (**vr**) of the root (see Boomsma & Sotiropoulos, 2016). Consequently, other factors, such as friction drag, which takes into account the effects of multiple grouped denticles (Dean & Bhushan, 2010), could not be fully achieved. In fact, the full mechanism of how dermal denticles act in hydrodynamics are not entirely understood, with subsequent works that have used different approaches proposing different results (Boomsma & Sotiropoulos, 2016). Nevertheless, all works that have explored the functionality of dermal denticles considered only fast‐swimming pelagic sharks, and none investigated slower‐swimming sharks, like the nurse shark. Consequently, interpretations available in literature about slower‐swimming sharks are only provided by Bechert et al. (1985) and Reif (1985).

However, we propose here a different interpretation for some denticles found in the nurse shark. We believe that at least part of the denticles in this species may have a contribution in hydrodynamics considering the distribution of ridged denticles in the animal. Ridged denticles combined with diamond or leaf‐shaped crowns are usually found in areas that would be associated with the drag reduction, like all the fins as well as dorsal and lateral regions of the trunk. Additionally, ridged denticles also found in the branchial tegument (Figure 4.3), may be associated with water flow in the branchial region as a reflection of the animals’ breathing mechanism and suction feeding of prey.

On the contrary, unridged denticles that are frequently associated with abrasion protection were also found in the head and ventral regions of the body. According to Reif (1985), unridged denticles are normally associated with other functions than hydrodynamics, with few exceptions like when present in the rostrum of fast pelagic species (e.g., *I. oxyrinchus*, *C. carcharias*). In the nurse shark, for instance, most unridged denticles are also the most robust, with a nearly lozenge‐shaped crown, peduncle and root, which may indicate the sole function of these denticles for abrasion protection. The distribution of these denticles may also support this interpretation, considering that the head and the ventral region of the body are areas that the animal usually uses to interact with the environment. For instance, nurse sharks are known for resting on the sandy bottom or in caves during daytime and to actively search for prey on the ocean floor at night (Ebert et al., 2021), which means that all ventral portions of the animal would be in constant friction with its environment. The hunting habits of this species also include active frictions of the entire head against small rocky reef spaces in search of hidden invertebrates and fish preys (Wilga et al., 2007). Parton et al. (2022) recently reported an upside down hunting pattern of the nurse shark which can explain the robust morphology of unridged denticles found in the dorsal region of head (Figure 4.1), and also of the robust ridged denticles found in type 1 dorsal denticles (Figure 5.1). Additionally, despite presenting ridges, oropharyngeal denticles seem to have a major function associated with abrasion protection as ridges aren't antero‐posteriorly oriented. This is consistent with the feeding type of the nurse shark, which consists mainly of harsh invertebrate prey (Ebert et al., 2021; Wilga et al., 2007). Ridges on the crown may also direct water flow around the taste bud (Atkinson & Collin, 2012).

Although the distribution and morphology of the placoid scales may also support functions other than abrasion strength, and may even indicate that ridged denticles may be hydrodynamic, only future studies using practical models will fully elucidate this hypothesis. However, we suggest that future works exploring dermal denticle morphology investigate different regions of the body, irrespective of the ecology and biology of the species, following recent approaches that have been applied only to fast pelagic sharks, but that were not further elaborated by Bechert et al. (1985) and Reif (1985) for slower‐swimming shark species. Many other slower taxa are known to bear ridged denticles (e.g., *M. canis* in Ankhelyi et al., 2018; Fath et al., 2024), so it is necessary to further investigate the possible function of ridged denticles and why they aren't exclusive for fast‐swimming sharks. It is also important to evaluate if Reif's interpretation for the functionalities of denticles in slow‐swimming sharks is still up‐to‐date after nearly 40 years. We also suggest that in depth studies of dermal denticle variation and morphology should not be restricted only to fast‐swimming sharks. We believe that a better understanding of morphological variation in denticles in slow‐swimming and bottom‐dwelling sharks may bring further contributions not only to studies on their functionality, but may also aid in future taxonomic and systematic analysis of the whole group (e.g., Vaz et al., 2023).

### 4.3 Dermal denticles as diagnostic characters

**FIGURE 11:**
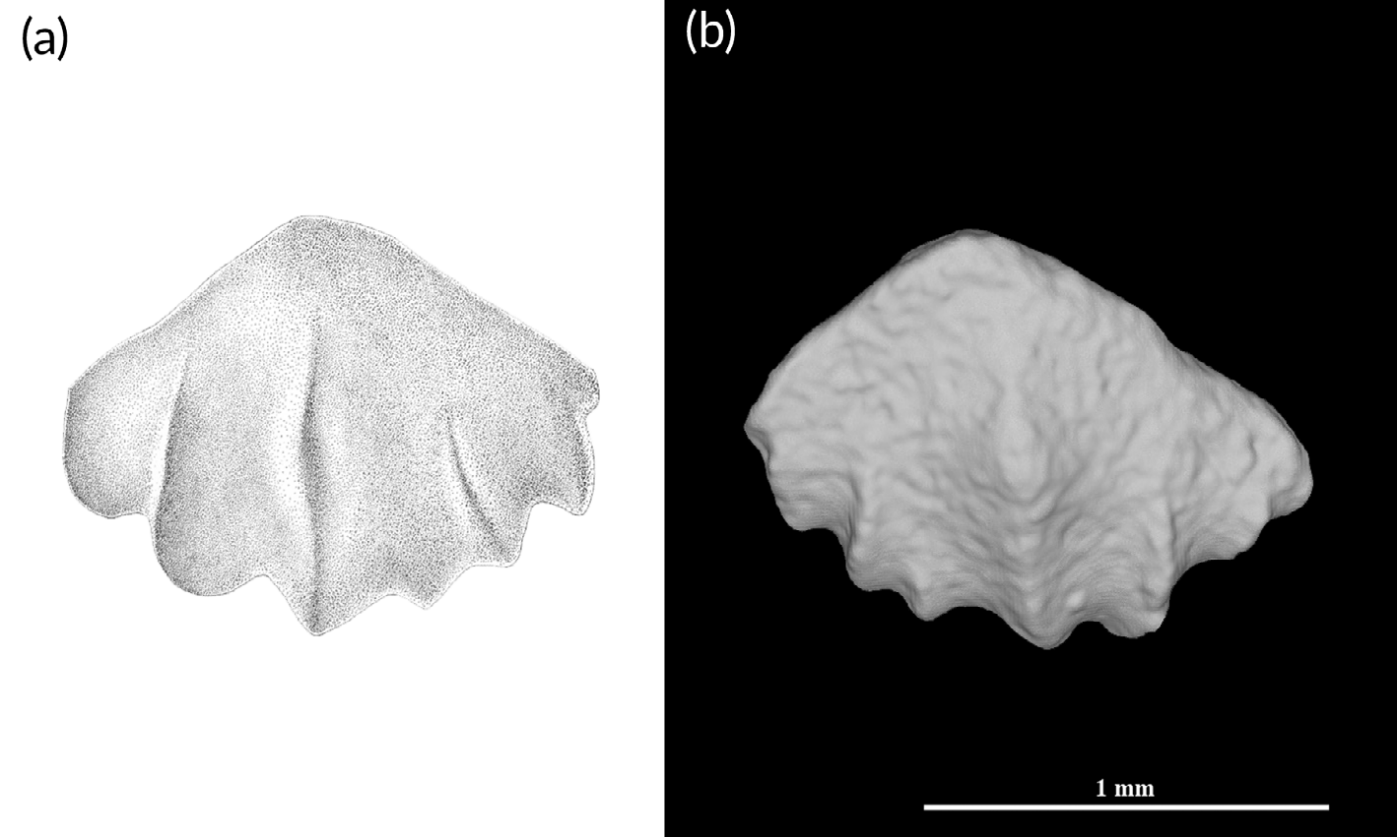
(a) Illustration of the denticle of the holotype of *Ginglymostoma unami* adapted from Del Moral‐Flores et al. (2015) with permission. (b) Micro‐CT scan of a D2 denticle described in the present work for *Ginglymostoma cirratum*. In (a, b) anterior to bottom.

The nurse shark *G. cirratum* was considered to be monotypic until 2015, when Del Moral‐Flores et al. (2015) described a new species for the genus. The species named *G. unami* is restricted to the East Pacific and was previously considered to be a putatively different population of the nurse shark. Among a wide variety of diagnostic characters that differentiate both species, one presented by the authors was the variable morphology of dermal denticles. Del Moral‐Flores et al. (2015) described the denticles of *G. unami* as being rhombic and bearing five to six ridges (Figure 11a) and that of *G. cirratum* as more elongated and with a smaller number of ridges. Moreover, the sampled regions for *G. unami* and *G. cirratum* were not indicated.

In the present study, a great amount of morphological differences and ridge countings were found. For instance, depending on the sampled region, ridges were absent or varied from one to nine, contrasting with Del Moral‐Flores et al. (2015). In our study, our total ridge counting number clearly surpasses the total ridge number reported by these authors for both species. Additionally, denticle morphology greatly varies along the body of the nurse shark. However, it is unclear if the same regions of the body were sampled in both species which may preclude an accurate comparative analysis.

The denticles depicted in Del Moral‐Flores et al. (2015) for *G. unami* are similar in shape to the ones found in the D2 sample of our work for *G. cirratum*. In this region, the crown morphology is very similar between the two species (described as a diamond shape in the present work), and the ridge count for *G. cirratum* varies from five to seven (Figure 11b). This falls into the range of ridges described to occur exclusively in *G. unami*. Consequently, denticle morphology would vary little between species and would not be reliable in this very specific case to discriminate and diagnose both species. This is in accordance with what is seen in other shark taxa, in which denticle morphology is usually correlated with ecological guild, and may consequently vary very little among species. This makes denticle identification difficult beyond family level for most cases (Dillon et al., 2017). For instance, Mello et al. (2013) discouraged species‐specific denticle identification in two families of Carcharhiniformes (Sphyrnids and Carcharhinids) considering the large amount of similarities they have found for denticles between different species.

To conclude, because the regions of the body sampled for *G. unami* are uncertain and its denticle morphology falls within the range of variation found for *G. cirratum*, it may be imprecise to use the denticle morphology as a diagnostic character. Consequently, we suggest that the regions of the body on any species of elasmobranch that are sampled to describe denticle morphology, and which may be used as a diagnostic character, are clearly defined in future taxonomic studies.

### 4.4 Final considerations

We have explored new morphological variations for the placoid scales in *G. cirratum*. It involved the sampling of 24 different regions of the body of three specimens of the nurse shark. However, we understand that the analysis of additional specimens of any species only strengthens any morphological finding. Nevertheless, we also acknowledge that most species of elasmobranchs are vulnerable and the number of specimens that can be collected or which are available for destructive dissections in fish collections are growingly scarce.

## AUTHOR CONTRIBUTIONS

Conceptualization: D. P. L., A. N. P. and J. P. C. B. S. Dissection of samples: D. P. L., A. N. P., L. F. M. and J. M. Micro CT scan and SEM: D. P. L., A. N. P., N. M. R. A., F. B. S and J. P. C. B. S. Data analysis and interpretation: D. P. L, A. N. P. and J. P. C. B. S. Drafting of the manuscript: D P. L. Critical revision of the manuscript and editing: D. P. L., A. N. P., L. F. M., J. M., M. V. G. A. and J. P. C. B. S. Approval: D P. L., A. N. P, L. F. M., N. M. R. A., J. M., M. V. G. A., F. B. S. and J. P. C. B. S.

## FUNDING INFORMATION

Fundação de Apoio à Pesquisa do Estado da Paraíba, grant number: 2019/2023; Conselho Nacional de Desenvolvimento Científico e Tecnológico (CNPq) grant number 142174/2018‐1; Coordenação de Aperfeiçoamento de Pessoal de Nível Superior (CAPES), grant numbers 88,887.912453/2023‐00; 88,887.670381/2022‐00; 88,887.670349/2022‐00; 88,887.987846/2024‐00; doctored grant‐financial code 001. Fundação de Amparo à Pesquisa do Estado de São Paulo (FAPESP) grant numbers 2010/52677‐6, 2012/22692‐9, 2024/14599‐6; Public Call n. 03 Produtividade em Pesquisa PROPESQ/PRPG/UFPB proposal code PIA13484‐2020.

## Data Availability

Micro CT‐scan images are available from the corresponding author upon reasonable request.
